# The relationship between quality of life and sex role of women with gynecological cancers undergoing brachytherapy

**DOI:** 10.1007/s00520-024-08559-3

**Published:** 2024-05-20

**Authors:** Esin Cerit, Dilek Efe Arslan, Dicle Aslan

**Affiliations:** 1 Yozgat Bozok University, Faculty of Health Sciences, Yozgat, Turkey; 2 Halil Bayraktar Health Services Vocational College, University of Erciyes, Kayseri, Turkey; 3 Department of Radiation Oncology, Faculty of Medicine, Erciyes University, Kayseri, Turkey

**Keywords:** Gynecologic cancer, Brachytherapy, The quality of life, The role of feminine

## Abstract

**Purpose:**

This study aims to analyze the relationship between the quality of life and sex roles of women diagnosed with cancer and undergoing brachytherapy.

**Methods:**

The research is a cross-sectional descriptive study. The sample of the study included 116 women over 35 years old who were diagnosed with a gynecologic cancer and underwent intracavitary brachytherapy at the Radiation Oncology Department of a university hospital. Personal information form, SF-36 the Quality of Life Scale, and BEM Sex Role Inventory were used in the study. The researcher collected the data through face-to-face interview. The data were collected in the nurses’ room after 3 different brachytherapy treatments that patients received weekly.

**Results:**

It was found that the average score of the physical functioning subscale was 32.80 ± 24.33, the average score of role physical was 15.43 ± 28.78, the average score of role emotional was 17.81 ± 28.96, the average score of vitality was 39.13 ± 16.12, the average score of social functioning was 43.53 ± 20.55, the score average of pain was 50.0 ± 20.09, the average score of general health was 42.67 ± 14.61, and the general health of mental health was 55.86 ± 16.12. In the BEM sex roles scale, the average score of BEM femininity was 105.56 ± 13.95, and the average score of BEM masculinity was 80.61 ± 12.77. In our study, a very low, negative, and significant relationship was determined between the role of femininity and emotional role limitation, physical functionality, social functionality, and general health perception in the women undergoing brachytherapy (*p* < 0.05).

**Conclusions:**

Based on the findings of the present study, we can state that an increase in the “role of femininity” in women undergoing brachytherapy was effective in the decrease in the quality of lives of women. It can be claimed that the results will be a guidance for the nurses who will play an important role in increasing the quality of lives of the women undergoing brachytherapy.

## Introduction

Despite the improvements in science and technology all over the world, cancer is one of the most important health problems today. With the improvement in the diagnosis facilities and the increase in the consciousness as well as opportunities for benefiting from healthcare institutions, more cancer cases are diagnosed every year [1]. The cancer statistics of the International Agency for Research on Cancer of the World Health Organization (IARC) indicated that there were an estimated 19.3 million new individuals with cancer in 2020 [2].

Parallel with the increase in the population in Türkiye, a similar increase is also observed in cancer cases. When the population of Türkiye became 81,916,866 in 2018, the number of new cases was reported as 210,537. Moreover, when the population of Türkiye became 84,339,067 in 2020, the number of new cases was reported as 233,834 [2]. The incidence of cancer has increased, and the types of cancer also vary depending on gender. The first 5 cancer types seen in women in our country are similar to the data of many countries in the world, and it has been determined that these are breast, thyroid, colorectal, uterine corpus, and respiratory cancers, and other gynecological cancers constitute 11.2% of all female cancers [1]. According to data from the International Agency for Research on Cancer (GLOBOCAN, 2020), gynecological cancers constitute 14.4% of new cancer cases in women worldwide [3]. Cervical cancer, ovarian cancer, and uterine cancer are the most common gynecological cancers in women [4, 5]. The most common cancer types in women of all age groups in Türkiye are known as breast cancer, thyroid cancer, and colorectal cancer; uterine cancer is the fourth, ovarian cancer is the seventh, and cervical cancer is the ninth most common cancer type. The fact that 3 gynecological cancers are among the top 10 most common cancer types in women reveals the importance of early diagnosis and treatment [1]. Gynecological cancers are malign diseases of female genital organs. Due to the increase in the incidence of gynecological cancers, brachytherapy, which is necessary for the treatment of these cancers, also becomes important [4, 6]. Brachy is the prefix meaning short, and therapy means treatment. The most important advantage of brachytherapy is that it ensures a high dose to be implemented in a localized area [6, 7]. In addition to the conveniences of the implementation of brachytherapy, the radiation dose applied during the treatment ceases ovary functions and causes irreversible sterility, and problems such as fatigue, nausea, and urinary incontinence can occur together with erythema, inflammation, mucosal atrophy, loss of elasticity in the vaginal epithelium, and ulceration problems in vaginal tissue [8, 9]. Gynecologic cancers and their treatments affect women’s communication with their partners, body image, sexual activity frequency, sexual response cycle, and quality of life in a negative way [8–11]. In the study conducted by Reb and Coge (2019) in California, it was found that the quality of life of the patients with gynecologic cancer was at a moderate level (6.4 ± 1.6) [12]. The quality of life might be described as the difference between the expectancies of individuals from life and what they achieve. The smaller the difference, the higher the quality of life [12–15]. However, one of the effects of the decrease in the quality of life is the gender role assigned to women [16, 17]. In the study carried out by Altın (2014), in order to analyze the personality characteristics of the women in our country in terms of the gender role they adopted, it was reported that women who adopted feminine gender roles were more tolerant, calm, and acquiescent, but they were emotionally unstable and unconfident, and those who adopted masculine gender roles were extroverted and sociable but less acquiescent and less calm [18].

In light of these data, the present study aims to analyze the relationship between the quality of lives of the women diagnosed with gynecologic cancer and undergoing brachytherapy and their gender roles. The number of studies focusing on women undergoing brachytherapy treatment is limited, and the fact that there is no other study handling gender role and the quality of life increases the importance of the study. This study might be a guidance for healthcare professionals to understand and solve the problems encountered by the women undergoing brachytherapy treatment and improve new care processes that can affect the quality of life positively.

## Methods

### Participants

The research is a cross-sectional descriptive study. The population of the study included 116 women over 35 years old and undergoing intracavitary brachytherapy in the Radiation Oncology Department of a university hospital. All women meeting the inclusion criteria and volunteered to participate in the study were included in the study. The post hoc power of the sample was determined based on the result of the post hoc analysis practiced by using study results on the G power* 3.1.9.4 statistical software. The power of the study, whose effect size was determined as 0.50 (large effect size ≥ 50) according to the correlation analysis (*r* − 0.187) performed between the score averages of SF-36 and BEM Sex Roles Inventory, was calculated as 99% [19].

#### Inclusion criteria

The inclusion criteria in the study were as follows:

- Do not have a physical or mental illness that would prevent them from answering the questions in their illness history in the online registration system in Turkey.
- Having cognitive abilities to understand and answer the questions.
- To be able to speak and understand Turkish.
- Diagnosed with non-metastatic endometrial or cervical cancer with or without external beam radiotherapy (EBRT).
- Completing regular brachytherapy treatment.
- The patients who undergo brachytherapy under anesthesia were not included in the study**.**

### Treatment features

Brachytherapy was applied to all patients in the same clinic. Adjuvant EBRT, chemotherapy, and surgical treatment options were left to the decision of the follow-up and treatment center. In patients who underwent EBRT, radiotherapy was applied to the pelvic lymph nodes and tumor bed at a dose of 1.8 Gy per fraction, for a total dose of 45 Gy. While a dose of 6 Gy was given per fraction in patients receiving EBRT in brachytherapy; patients who did not receive EBRT received a dose of 7 Gy per fraction. EBRT was administered using either intensity-modulated radiotherapy (IMRT) or volumetric modulated arc therapy (VMAT) techniques were used in EBRT. Three-dimensional radiotherapy (3D-RT) technique was applied in brachytherapy in all patients. In brachytherapy, the procedure was performed by selecting one of the cylinder or ovoid applicators (Varian Medical Systems, Palo Alto, CA, USA) suitable for the patient’s anatomy.

### Procedures

Before the study, all patients were informed about the study, and their written consents were received. The participants received their brachytherapy treatments between 09:00 and 12:00. Brachytherapy was applied on Mondays, Wednesdays, and Fridays every week. The researcher visited the Radiation Oncology Department 3 days a week, received the patient list from the nurse, and patients whose brachytherapy treatments finished were called in the nurses’ room, respectively. The data were collected through face-to-face interviews lasting about 20 min.

### Measures

The data were collected by using the “Personal Information Form,” which was created through a literature review, BEM Sex Role Inventory, and SF-36 the Quality of Life Scale.

#### Personal information form

The Personal Information Form was created based on a literature review by the researchers, and it included questions related to the socio-demographic characteristics (age, marital status, number of children, family type, educational status, occupation, marriage duration, income, working status, and health insurance type) and health states (smoking and alcohol use, diagnosis stage, diagnosis period, the state of the disease, treatment type, and undergoing operation) of the women [9, 18, 20, 21].

#### BEM Sex role inventory

The inventory developed by BEM (1974) and adapted into Turkish by Kavuncu (1987) was used. The items in the inventory represent 40 items including 20 adjectives related to males and 20 adjectives related to females. The scale is a 7-point Likert scale including the options of “never true,” “usually not true,” “sometimes not true,” “undecided,” “sometimes true,” “usually true,” and “always true.” The scale yields two types of scores: femininity and masculinity. Based on the medians of these scores, 4 gender roles are identified: feminine, masculine, androgynous, and undifferentiated. Femininity score, those scoring above the femininity median and below the masculinity score median are categorized as “feminine”; masculinity score, those scoring above the masculinity median and below the femininity score median are categorized as “masculine”; individuals with scores above both the femininity and masculinity medians are considered “androgynous”; those with scores below both the femininity and masculinity medians are classified as “undifferentiated” gender role. The participants are asked to mark to what extent each adjective describes them. In the Turkish version of the scale, feminine traits are described as “virtuous, affectionate, dignified, serious, self-sacrificing, emotional,” while masculine traits include “generous, trustworthy, courageous, responsible towards family, authoritative, rational, not expressing emotions, rule-oriented, strict, and idealistic.” The high-reliability coefficients of the femininity (0.75) and masculinity (0.89) subscales used in the inventory suggest that the instrument can be considered a reliable inventory [18, 21–23].

#### SF-36 the quality of life scale

SF36 the Quality of Life Scale was developed by Rand Corporation in 1992. The Turkish validity and reliability study of the scale was conducted by Koçyiğit et al., and the scale was found valid and reliable for the Turkish context. The scale includes 36 items and eight subscales. The physical functioning subscale includes 10 items, role physical 4 items, bodily pain 2 items, general health 5 items, vitality 4 items, social functioning 2 items, role emotional 3 items, and mental health 5 items. In addition, the changes in the health state compared to the previous year are asked in the second item. The minimum score that can be obtained from the scale is 0, the maximum is 100, and as the score increases the quality of life also increases. The total score average of the scale is not calculated [24].

### Data analysis

SPSS 23.0 program was used for data analysis in the study. Digit (*n*), percentage (%), mean, and standard deviation (SD) were used as descriptive statistical methods. The compliance of the data with normal distribution was evaluated by using the Shapiro–Wilk test and QQ diagrams. The relationship between the scale scores was examined through Pearson correlation analysis. The effect of the scale score averages on each other was determined by using linear regression analysis. Linear regression analysis has been used to assess the influence of participants’ gender roles on their levels of quality of life. The statistical significance for all analyses was found as *p* < 0.05.

## Results

The data collection in the study was conducted through face-to-face interviews with voluntary individuals who met the study criteria, ensuring no data loss. The data of all participants initially included in the research (116 individuals) were utilized.

Table 1 lists the descriptive characteristics of the participants (Table 1).

**Table 1:** The descriptive data of the personal characteristics of the participants

Characteristics	*n*	%
Age (*X* ± *SD years)*	61.48 ± 9.01	
38–64	72	62.1
65 and over	44	37.9
Marital status		
Married	100	86.2
Single	16	13.8
Family type		
Nuclear family	72	62.1
Extended family	44	37.9
Education status		
Illiterate	53	45.3
Literate	38	32.5
Primary education	25	22.2
Place of residence		
A city center	87	75.7
Town	22	19.1
Village	7	5.2
Smoking status		
No	116	100.0
Drinking status		
No	116	100.0
Diagnosis		
Endometrium CA	93	80.2
Cervix CA	23	19.8
Stage of the disease*		
1a	47	50.5
1b	38	40.9
2a	4	4.3
2b	4	4.3
3a	0	0.0
Stage of the disease**		
1a	0	0.0
1b	11	47.8
2a	7	30.4
2b	3	13.0
3a	2	8.7
Operated for their diagnoses		
No	21	18.1
Yes	95	81.9
Chemotherapy		
No	89	76.7
Yes	27	23.3
External beam radiotherapy		
Yes	20	17.3
No	96	82.7
Total	116	100.0

*X*, mean; *SD*, standard deviation

^*^Endometrium CA

^**^Cervix CA

Table 2 presents the mean scores for the BEM Sex Role Inventory and SF-36 the Quality of Life Scale (Table 2).

**Table 2:** The distribution of scores obtained by patients from the subscales of both the SF-36 Quality of Life Scale and the BEM Sex Roles Scale (n:116)

	*X* ± SD (median)	Min–max
SF-36 the Quality of Life Scale		
Physical functioning	32.80 ± 24.33(30)	0.00–95.00
Role physical	15.43 ± 28.78(0)	0.00–100.00
Role emotional	17.81 ± 28.96(0)	0.00–100.00
Vitality	39.13 ± 16.12(40)	5.00–80.00
Social functioning	43.53 ± 20.55(37.5)	0.00–100.00
Pain	50.0 ± 20.09(55)	12.50–100.00
General health	42.67 ± 14.61(40)	5.00–80.00
Mental health	55.86 ± 16.12(56)	20.00–100.00
BEM Sex Roles Scale		
BEM femininity	105.56 ± 13.95 (106)	65.00–134.00
BEM masculinity	80.61 ± 12.77 (82)	44.00–108.00

*X*, mean; *SD*, standard deviation; *Min*, minimum; *Max*, maximum

Table 3 contains the distribution of the participants regarding sex roles determined in line with the BEM feminine median value (106) and the BEM masculine median value (82) (Table 3).

**Table 3:** Distribution of participants according to sex roles

Sex roles*	*n*	%
Femininity	26	22.41
Masculinity	28	24.13
Androgynous	21	18.10
Undifferentiated	41	35.34

^*^It was calculated by taking into account the median values of BEM femininity and BEM masculinity

According to Table 4, there is a negative, very low level of significant relationship between BEM femininity roles and physical functioning (*r* − 0.187), general health (*r* − 0.185), social functioning (*r* − 0.186), and role emotional (*r* − 0.210) (*p* < 0.05). There is no statistically significant relationship between BEM femininity scale and the other subscales of SF-36 scale (*p* > 0.05). No statistically significant relationship was found between the averages of BEM masculinity role and subscale score of SF-36 the Quality of Life Scale (*p* > 0.05). There is no statistically significant relationship between the averages of BEM masculinity role and femininity role scores (*p* > 0.05) (Table 4). The degree of relationship between variables can be interpreted as weak when the correlation coefficient is between 0 and 0.29, moderate when it is between 0.30 and 0.64, strong when it is between 0.65 and 0.84, and very strong when it is between 0.85 and 1 [25].

**Table 4:** The Relation between SF 36 the Quality of Life Subscale Score averages and BEM Sex Roles Scale Score averages

Correlations		Physical functioning	Role physical	Pain	vitality	Social functioning	Role emotional	Mental health	General health	Age
*BEM femininity*	*r*^*a*^	− 0.187	− 0.140	0.176	− 0.091	− 0.186	− 0.210	0.047	− 0.185	− 0.030
	*p*	***0.046***	0.136	0.061	0.330	***0.046***	***0.024***	0.617	***0.047***	0.750
*BEM masculinity*	*r*^*a*^	0.849	− 0.051	0.096	− 0.048	0.041	− 0.010	0.032	0.039	− 0.071
	*p*	0.113	0.590	0.309	0.612	0.662	0.918	0.715	0.682	0.449

*r*^*a*^*: Pearson correlation analysis applied*

Table 5 contains the findings of the effects of the scores the participants obtained from the BEM Sex Roles Scale on the average scores of the subscales of SF-36 the Quality of Life Scale. According to Table 4, the feminine gender role of women significantly affects physical functionality by 3.5%, role emotional by 4.4%, social functionality by 3.4%, and general health by 3.4%. In addition, it has been found that the increase in the femininity gender role of the participants decreased the physical functionality level by 0.187 times, the social functionality by 0.186 times, the role emotional by 0.210 times, and the general health by − 0.185 times (Table 5).

**Table 5:** The effect of the score averages of BEM Scale Femininity Role on SF-36 Scale Score averages

Model		B	Standart error	Beta	*T*	*p*
*BEM femininity*	Physical functioning *R* = 0.187, *R*^2^ = 0.035 *F* = 4.055, *p* = 0.046	67.515	17.38	− 0.187	3.884	***0.046***
	Social functioning *R* = 0.186, *R*^2^ = 0.034 *F* = 4.071, *p* < 0.001	72.418	14.43	− 0.186	− 2.018	***< 0.001***
	Role emotional *R* = 0.210, *R*^2^ = 0.044 *F* = 5.240, *p* = 0.024	63.772	20.24	− 0.210	3.149	***0.002***
	General health *R* = 0.185, *R*^2^ = 0.034 *F* = 4.036	63.127	10.26	− 0.185	6.147	***0.047***

^*^Linear regression analysis was used

## Discussion

Nowadays, an increase in brachytherapy use is observed since it provides a plethora of advantages such as precise and target-specific features, few side effects compared to other treatment methods, decrease in treatment durations, and cost efficiency [26]. Despite all these advantages, it is emphasized in the studies dealing with the side effects of brachytherapy that it has several side effects such as pain, impairment in sexual functionality, stigmatization and impairment in self-respect, embarrassment because of intervention in an intimate region, and body image that may impair women’s quality of lives [20, 27–30]. The relationship between the gender roles of the women undergoing brachytherapy and the quality of life was analyzed in this study. It is a well-known fact that the judgments for the gender roles adopted in societies are one of the most significant factors for individuals to adapt to their lives [31]. In our study, a negative and very low level of significant relation was found between feminine gender role of women undergoing brachytherapy and emotional role, physical functionality, social functionality, and general health. In the study, it was found that the feminine gender role of women significantly reduced their physical functionality, emotional role restriction, social functionality level, and general health perception, albeit to a small extent. There is no study analyzing the effect of having feminine gender roles of women undergoing brachytherapy treatment on their quality of life in the literature. However, there are a limited number of studies examining the relationship between individuals’ gender roles and their perceptions of quality of life and illness. In a study conducted by Pikler and Brown (2010) on 300 cancer patients, it was determined that gender role identities had no effect on quality of life, but individuals who attributed a masculine or undifferentiated gender role identity had a higher risk of developing depressive symptoms compared to those who attributed a feminine or androgynous gender [32]. Moreover, in a study conducted by Willerth et al. on 2002 individuals, the femininity role was associated with poorer self-rated health [33]. Results vary from studies in the literature. These results suggest that this is due to the difference in societies’ perspectives on the roles of men and women. For example, in our country, where the patriarchal traditional structure is still dominant, the studies conducted on the “feminine” role perception highlight that women contend that their roles in life are giving birth to children, meeting the needs of their husbands and children, ensuring the unity of the family, supporting their husbands, etc. In this context, while the dominance of the feminine role brings tolerance, calm, and being acquiescent, it reveals a lack of self-confidence and unbalance emotionally in women. In this context, especially for women who have a more feminine gender role, feeling inadequate, the thought of losing their feminine role, and the negative emotions caused by constant intervention in an intimate region lead to much more negative situations in women’s lives. We can say that this negative process causes women to have difficulty coping with the side effects of the treatment and to a decrease in their quality of life [34, 35]. It is inevitable that the side effects of brachytherapy, including sexual functionality, stigmatization, and deterioration in body image and self-esteem, prevent women from fulfilling their gender roles. Not only undergoing brachytherapy but also the diagnosis of gynecological cancer itself has an impact on sex roles in women. Marifah et al. (2024) in their study of Indonesian women diagnosed with gynecological cancer stated that Indonesian women are strongly committed to traditional femininity roles and that they prioritize their perceived duties towards their husbands within the sex role over their own rights. As a result of this qualitative study, it was stated that even if patients recover from gynecological cancer, sexual dysfunction develops and the source of this problem is traditional sex roles [36]. In the study conducted by Uceda-Escobar et al. (2023) on women with gynecological diagnoses who had radiotherapy, it was shown that femininity, fertility, and femininity roles were negatively affected due to changes in the body [37]. In the same study, it was observed that women experienced feelings of shame by using submission, spirituality, and emotional avoidance to combat this situation. This feeling of shame and avoidance was thought to be due to the secondary effect of radiotherapy treatment. In this regard, it is an expected result that the quality of life of the women who think that they cannot fulfill their femininity roles is negatively affected by this. In Türkiye, nurses are the health professionals who are in most contact with patient women and their families during the brachytherapy process. In this context, nurses working in radiation oncology should provide and implement care coordination to support the treatment of the patient, taking into account the sex roles of the patients in their own cultural and belief systems, in the light of evidence-based guidelines aimed at improving the quality of life of patients, provide emotional support and motivation to treatment, provide counseling, organize trainings and conduct individual patient-oriented interviews [38].

## Conclusion

As a result, brachytherapy is a treatment process that negatively affects the lives of patients and their relatives, despite the many conveniences it provides in the treatment process of gynecological cancers. During this difficult process, multidisciplinary and holistic care must be provided to the patient and her family. Nurses are the health professionals who are in most contact with patient women and their families during the brachytherapy process. In this process, in order to minimize the deterioration in women’s quality of life due to brachytherapy, the nurse must have sufficient knowledge and skills about the effect of brachytherapy on the lives of patients and be able to effectively evaluate the risk factors that will impair their quality of life. In this context, nurses should be aware of individual needs and plan care accordingly, establish more open communication with patients to allow them to express their feelings regarding changes in their bodies, provide clear information to patients and their families about the side effects and coping methods of brachytherapy, offer understandable recommendations on daily life activities such as clothing selection, genital hygiene, sexual intercourse, etc., and suggest expert support for psychological counseling based on the individual’s needs.

Given the findings of the present study, we can state that the increase in the “femininity role” in women undergoing brachytherapy is effective in reducing the quality of life. The fact that the number of studies analyzing the effect of brachytherapy application, which is an efficient treatment method in gynecologic cancers, on women’s lives is limited increases the importance of our study since the study results will guide future studies, and our study has also revealed that there is a huge gap in the literature.

### Limitations

The study had certain limitations. Firstly, it was conducted in only one center. Therefore, the results of the study cannot be generalized to other patients undergoing a different brachytherapy protocol. The physical and psychosocial symptoms of the women in the treatment group were not analyzed. The women undergoing brachytherapy under anesthesia, which was thought to influence the answers of the patients, were not included in the study.

## Data Availability

The data that support the findings of this study are available on request from the corresponding author. The data are not publicly available due to privacy or ethical restrictions.

## References

[1] Türkiye Cancer Statistics. T.R. Ministry of Health Türkiye General Directorate of Public Health Cancer Department. (2016) hsgm.saglik.gov.tr/tr/kanser-istatistikleri/yillar/2016-yili-turkiye-kanser-ihttps://statistikleri.html. Accessed 10 Aug 2023

[2] International Agency for Research on Cancer (IARC) (2023) https://www.iarc.who.int/branches-csu/. Accessed 10 Sept 2023

[3] Sung H, Ferlay J, Siegel RL, Laversanne M, Soerjomataram I, Jemal A, Bray F (2021) Global cancer statistics 2020: GLOBOCAN estimates of incidence and mortality worldwide for 36 cancers in 185 countries. CA: Cancer J Clin 71(3):209–249 10.3322/caac.21660 33538338

[4] Yi M, Li T, Niu M, Luo S, Chu Q, Wu K (2021). Epidemiological trends of women’s cancers from 1990 to 2019 at the global, regional, and national levels: a population-based study. Biomarker Res.

[5] Rosa LM, Hames ME, Dias M, Miranda GM, Bagio CB, Santos MJ, Kalinke LP (2019). Epidemiological profile of women with gynecological cancer in brachytherapy: a cross-sectional study. Rev Bras Enferm.

[6] Ledford LR, Lockwood S (2019). Scope and epidemiology of gynecologic cancers: an overview. Semin Oncol Nurs.

[7] Annede P, Dumas I, Schernberg A, Tailleur A, Fumagalli I, Bockel S, Mignot F, Kissel M, Deutsch E, Haie-meder C, Chargar C (2019). Radiobiological optimization comparison between pulse-dose-rate and high-dose-rate brachytherapy in patients with locally advanced cervical cancer. Brachytherapy.

[8] Corrêa CSL, Leite ICG, Andrade APS, Guerra MR (2015). Cervical cancer treatment and its effects on sexual function: recent evidence and approach. Austin J Womens Health.

[9] İnel S (2018) Radyoterapi ve Brakiterapide Yan Etkiler. CBU-SBED: Celal Bayar Univ-Health Sci Ins J 5(1):17–19

[10] Barcellini A, Dominoni M, Dal Mas F, Biancuzzi HA, Venturini SC, Gardella B, Orlandi E, Bø K (2022) Sexual health dysfunction after radiotherapy for gynecological cancer: role of physical rehabilitation including pelvic floor muscle training. Front Med 8:2821 10.3389/fmed.2021.813352 PMC8852813 35186978

[11] Warnock C (2009) Patients’ experiences of ıntracavity brachytherapy treatment forgynaecological cancer. Eur J Oncol Nurs 9(1):44e55 10.1016/j.ejon.2004.03.009 15774340

[12] Reb AM, Cope DG (2019). Quality of life and supportive care needs of gynecologic cancer survivors. West J Nurs Res.

[13] La Rosa VL, Shah M, Kahramanoglu I, Cerentini TM, Ciebiera M, Lin LT, Dirnfeld M (2020). Quality of life and fertility preservation counseling for women with gynecological cancer: an integrated psychological and clinical perspective. J Psychosom Obstet Gynecol.

[14] Ashley L, Marti J, Jones H, Velikova G, Wright P (2015). Illness perceptions within 6 months of cancer diagnosis are an independent prospective predictor of health-related quality of life 15 months post diagnosis. Psycho Oncol.

[15] Haraldstad K, Wahl A, Andenæs R, Andersen JR, Andersen MH, Beisland E, Borge CR (2019). A systematic review of quality of life research in medicine and health sciences. Qual Life Res.

[16] Kissal A, Beser A (2011). Knowledge, facilitators and perceived barriers for early detection of breast cancer among elderly Turkish women. Asian Pac J Cancer Prev.

[17] Norris CM, Murray JW, Triplett LS, Hegadoren KM (2010). Gender roles in persistent sex differences in health-related quality-of-life outcomes of patients with coronary artery disease. Gend Med.

[18] Altın B (2014) Examination of personality traits in women according to gender roles. (Unpublished Master’s Thesis) Istanbul Bilim University Institute of Social Sciences, Istanbul. https://tez.yok.gov.tr/UlusalTezMerkezi/tezSorguSonucYeni.jsp. Accessed 10 Jul 2023

[19] Corpes EDF, Gonçalves GDA, Oliveira ACA, Pacífico VS, Castro RCMB, Almeida PC, Barbosa IM (2022). Effects of brachytherapy on qualıty of lıfe and functıonalıty ın the treatment of cervıx cancer. Cogitare Enfermagem.

[20] Kazoba F, Dharsee N (2020). Experiences of women receiving high dose rate brachytherapy for cervical cancer at ocean road cancer institute Dar-es-salaam Tanzania. Glob J Cancer Ther.

[21] Bem S (1993). The lenses of gender.

[22] Kavuncu NA (1987). Studies on adapting the bem gender role ınventory to Turkish Society (Published Master’s Thesis).

[23] Dökmen ZY (1999). BEM Cinsiyet Rolü Envanteri Kadınsılık Ve Erkeksilik Ölçekleri Türkçe Formunun Psikometrik Özellikleri. Kriz Dergisi.

[24] Kocyigit H, Gülseren Ş, Erol A, Hizli N, Memis A (2003). The reliability and validity of the Turkish version of Quality of Life Questionnaire of the European Foundation for Osteoporosis (QUALEFFO). Clin Rheumatol.

[25] Ural A, Kılıç İ (2013). Bilimsel Araştırma Süreci ve SPSS ile Veri Analizi.

[26] Banerjee R, Kamrava M (2014). Brachytherapy in the treatment of cervical cancer: a review. Int J Women’s Health.

[27] Uysal N, Ünal Toprak F, Soylu Y, Kaya B (2020). Evaluation of sexual life in patients with gynecologic cancer undergoing radiotherapy. Sex Disabil.

[28] Faithfull S, White I (2008). Delivering sensitive healthcare information: challenging the taboo of women’s sexual health after pelvic radiotherapy. Patient Educ Coun.

[29] Wilson CM, McGuire DB, Rodgers BL, Elswick RK, Menendez S, Temkin SM (2020). Body ımage, sexuality, and sexual functioning in cervical and endometrial cancer: ınterrelationships and women’s experiences. Sex Disabil.

[30] Öztürk ÇS (2019) Qualitative determination of the physical and psychological effects of brachytherapy in patients receiving gynecological cancer treatment. (Unpublished Master’s Thesis) Istanbul University-Cerrahpaşa Graduate Education Institute, Istanbul. https://tez.yok.gov.tr/UlusalTezMerkezi/tezSorguSonucYeni.jsp. Accessed 15 Jul 2023

[31] Ballard-Reisch D, Elton M (1992). Gender orientation and the Bem Sex Role Inventory: a psychological construct revisited. Sex Roles.

[32] Pikler VI, Brown C (2010). Cancer patients’ and partners’ psychological distress and quality of life: influence of gender role. J Psychosoc Oncol.

[33] Willerth M, Ahmed T, Phillips SP, Pérez-Zepeda MU, Zunzunegui MV, Auai M (2020). The relationship between gender roles and self-rated health: a perspective from an international study. Arch Gerontol Geriatr.

[34] Buran K, Bektaş Ç (2022). Women’s gender role perceptions: a research on university students. Int Soc Mentality Res Thinkers J.

[35] Savaş G (2018). Gender (In) equality perception of individuals living in Turkey. Mediterr J Women’s Stud Gend.

[36] Ma’rifah AR, Afiyanti Y, Djatmiko W, Ruwaida I, Milanti A (2024). Gender role conflicts experienced by Indonesian women with gynecological cancer: a phenomenological study. Belitung Nurs J.

[37] Uceda-Escobar A, Guerra-Martín MD, Botello-Hermosa A (2023). The perceptions of women with gynecological cancer after radiotherapy treatment: a gender-based qualitative study. Healthcare.

[38] Rose P, Yates P (2013). Person centred nursing care in radiation oncology: a case study. Eur J Oncol Nurs.

